# Multiple Arterial Thrombosis Related with Cantharidin Ingestion

**DOI:** 10.1155/2018/8159093

**Published:** 2018-02-19

**Authors:** Zehra Narli Ozdemir, Ugur Sahin, Sinem Civriz Bozdag, Osman Ilhan

**Affiliations:** Department of Hematology, Faculty of Medicine, Ankara University, Ankara, Turkey

## Abstract

Inherited and/or acquired thrombophilic defects can result in venous or arterial thrombosis. This case report describes arterial thrombotic episodes triggered by the ingestion of an aphrodisiac remedy containing cantharidin in a 46-year-old female patient later discovered to be heterozygous for prothrombin G20210A mutation and seropositive for anti-*β*2 glycoprotein-I (anti-*β*2-GPI) antibodies of IgA isotype.

## 1. Introduction

The association between the risk of clinically apparent thrombosis and seropositivity for anti-phospholipid (aPL) antibodies including lupus anticoagulant (LA), anti-cardiolipin (aCL), anti-*β*2-GPI antibodies is well described [1]. Among the inherited thrombophilias, prothrombin G20210A mutation is the common and particularly related with increased risk for venous thrombosis [2].

Cantharidin, the main active component in preparations of *Lytta vesicatoria*-derived Spanish fly, was first isolated in 1810 and has been investigated in toxicology since then.

This paper aims to present a temporal association of thrombotic episodes with the ingestion of an aphrodisiac remedy containing cantharidin in an adult female patient later discovered to be heterozygous for prothrombin G20210A mutation and seropositive for anti-*β*2GPI antibodies of IgA isotype.

## 2. Case Presentation

A 46-year-old female patient was admitted to our outpatient clinic for further investigation of arterial thrombotic episodes. She had symptoms of dyspnea and chest pain which started two months ago, and prior evaluation with pulmonary computed tomography (CT) angiography had revealed acute thrombosis of right bronchial artery. Pain and weakness developed in the right arm within a few days, and the Doppler ultrasound demonstrated thrombosis of right radial artery. Two days after starting treatment with subcutaneous enoxaparin at a dose of 1 mg/kg bid and acetylsalicylic acid at a dose of 100 mg/day, she presented with severe abdominal pain, nausea, and vomiting. The abdominal CT scan revealed splenic infarction, which necessitated splenectomy.

Her past medical history was unremarkable for any chronic condition or medication use except a commercially manufactured solution of Spanish fly or *Lytta vesicatoria*, which she had been using for about six months to get an aphrodisiac effect. Her family history was also unremarkable except her father, who had died due to complications of mesenteric ischemia. Rheumatologic assessment and physical examination on admission were unremarkable otherwise.

Laboratory tests were as follows: white blood count: 12.05 × 10^9^/L; hemoglobin: 12.9 g/dL; platelet: 500 × 10^9^/L; blood urea nitrogen: 8 mg/dL; creatinine: 0.71 mg/dL; alanine aminotransferase: 29 U/L; aspartate aminotransferase: 22 U/L; lactate dehydrogenase: 167 U/L; prothrombin time (PT): 23.6 sec; activated partial thromboplastin time (aPTT): 34.9 sec; INR: 2.09 (secondary to warfarin); d-dimer: 71 ng/mL; plasma fibrinogen: 2.92 g/L; protein C: 71%; protein S: 83%; activated protein C resistance (APC): 2.74 R. During the follow-up, the patient had persistent mild thrombocytosis, which was presumed to be postsplenectomy thrombocytosis. Antinuclear antibody (ANA) was positive stained as dense fine speckled 70, and anti-neutrophil cytoplasmic antibody (ANCA) was negative. Thrombophilia screening test results were as follows: erythrocyte sedimentation rate: 8 mm/h; C-reactive protein: 2.2 mg/L and anti-dsDNA: 8.4 IU/mL; LA: 1.1 R; anti-CL antibodies of IgM isotype: 0.9 MPL·U/mL; IgG isotype: 0.9 GPL·U/mL; anti-phosphatidylserine antibodies of IgM isotype: 1.7 MPL·U/mL; IgG isotype: 1.7 GPL·U/mL; IgA isotype: 1.2 RU·U/mL; anti-*β*2-GPI IgM isotype: 2.2 MPL·U/mL; IgG isotype: 1.7 GPL·U/mL; IgA isotype: 146.2 U/mL. All these thrombophilia screening tests were repeated two months later, and all of them were in normal range except anti-*β*2-GPI antibodies IgA isotype. Anti-*β*2-GPI IgA levels were detected at second and fourth months, 201.7 U/mL and 122.4 U/mL, respectively. Factor V Leiden mutation was negative, and prothrombin G20210A mutation was heterozygote positive. Chronic myeloproliferative disorders were excluded with negative *BCR-ABL* and *JAK2 V617F* mutations and normal peripheral blood smear. Paroxysmal nocturnal haemoglobinuria clones were not detected by flow cytometric investigation. Echocardiographic findings and cardiologic assessment were considered normal and not associated with any source of thromboembolism. All these laboratory tests mentioned above were obtained in our clinic about two months after the first thrombotic episode while she was taking warfarin.

## 3. Discussion

In this case report, we present a patient who had arterial thrombosis and splenic infarct, also found to be heterozygous for prothrombin G20210A mutation and seropositive for anti-*β*2-GPI antibodies of IgA isotype.

Anti-phospholipid antibodies may predispose to thrombosis in venous and arterial circulation, as well as microcirculation in the absence of a defined anti-phospholipid antibody syndrome. However, not all aPL antibodies are thrombogenic, and transient increases in serum aPL antibodies have been reported during several infections [1]. Among otherwise healthy people, the incidence of clinically silent aPL antibody seropositivity ranges from 1 to 5% [1]. According to current data, the persistence of aPL antibodies in asymptomatic carriers conveys a risk factor for future thrombotic events, particularly among patients with double or triple positive test results [3].

Drug-induced lupus anticoagulant (DILA) is a well-known entity with heterogeneous features and various laboratory findings as well as related clinical complications. Despite the drugs associated with LAs are pharmaceutically not similar and heterogeneous group, one of the hypotheses that may explain the associations is disruption of the cell membrane. Drugs' pharmacologic action by perturbing cell membranes may decrease action potentials and propagation of charge that may facilitate adhesion of antigens, for example, *β*2GPI or prothrombin, on the cell surface and may induce latent or new antigens. The second hypothesis is about drug-induced autoimmunity which induces B-lymphocytes producing autoantibodies to self-proteins, including phospholipid-binding proteins such as *β*2GPI and prothrombin [4]. In our case, anti-*β*2GPI antibodies of IgA isotype may have appeared due to cantharidin ingestion, but we were not able to follow up antibody titers whether diminishing or not after the fourth month.

Prothrombin G20210A mutation may contribute to a state of hypercoagulability, but not particularly with arterial thrombosis including acute ischemic stroke or transient ischemic attack [2, 5]. Heterozygotes for the 20210A allele have a 2- to 5-fold increase in venous thrombosis risk compared with healthy control group [6].

Cantharidin has been used since ancient times as an aphrodisiac due to its effect of perceiving sexual arousal. Poisoning usually results after aphrodisiac ingestion [7]. Ingesting cantharidin can initially cause severe damage to the lining of the gastrointestinal and urinary tracts and may also cause acute tubular necrosis. Priapism, seizures, and cardiac abnormalities are less commonly seen [8]. Cantharidin poisoning manifests symptoms including lumbar pain, dysuria, proteinuria, abdominal pain, dysphagia, hematemesis, vomiting, and rarely prolonged erections [7]. In our case, first thrombotic event appeared approximately six months after the initiation of Spanish fly, and the patient has experienced no more thrombotic episodes since cessation of cantharidin ingestion.

We propose that cantharidin exerts its thrombotic effects via two possible major mechanisms. The first is its direct toxic effect on endothelial cells, which was reflected to previous reports as mucosal erosions and nephropathy [7–9]. The second mechanism, which is our original contribution, is the identification of anti-phospholipid antibodies in this patient's blood. Since prothrombin G20210A mutation is mainly reported to increase venous thrombosis risk, its presence in this case is considered as coincidental. However, it might also have contributed to the prothrombotic state together with accompanying mild thrombocytosis, as well.

In conclusion, to our knowledge this is the first case report about thrombotic events after ingestion of cantharidin in a patient with anti-*β*2-GPI and prothrombin G20210A mutation.
